# An Insight into the Sialotranscriptome of the Cat Flea, *Ctenocephalides felis*

**DOI:** 10.1371/journal.pone.0044612

**Published:** 2012-09-25

**Authors:** José M. C. Ribeiro, Teresa C. F. Assumpção, Dongying Ma, Patricia H. Alvarenga, Van M. Pham, John F. Andersen, Ivo M. B. Francischetti, Kevin R. Macaluso

**Affiliations:** 1 Vector Biology Section, Laboratory of Malaria and Vector Research, National Institute of Allergy and Infectious Diseases, Rockville, Maryland, United States of America; 2 Laboratório de Bioquímica de Resposta ao Estresse, Instituto de Bioquímica Médica, Universidade Federal do Rio de Janeiro, Rio de Janeiro, Brazil; 3 Instituto Nacional de Ciência e Tecnologia em Entomologia Molecular (INCT-EM), Rio de Janeiro, Brazil; 4 Department of Pathobiological Sciences, School of Veterinary Medicine, Louisiana State University, Baton Rouge, Louisiana, United States of America; Metabiota, United States of America

## Abstract

**Background:**

Saliva of hematophagous arthropods contains a diverse mixture of compounds that counteracts host hemostasis. Immunomodulatory and antiinflammatory components are also found in these organisms' saliva. Blood feeding evolved at least ten times within arthropods, providing a scenario of convergent evolution for the solution of the salivary potion. Perhaps because of immune pressure from hosts, the salivary proteins of related organisms have considerable divergence, and new protein families are often found within different genera of the same family or even among subgenera. Fleas radiated with their vertebrate hosts, including within the mammal expansion initiated 65 million years ago. Currently, only one flea species–the rat flea *Xenopsylla cheopis*–has been investigated by means of salivary transcriptome analysis to reveal salivary constituents, or sialome. We present the analysis of the sialome of cat flea *Ctenocephaides felis*.

**Methodology and Critical Findings:**

A salivary gland cDNA library from adult fleas was randomly sequenced, assembled, and annotated. Sialomes of cat and rat fleas have in common the enzyme families of phosphatases (inactive), CD-39-type apyrase, adenosine deaminases, and esterases. Antigen-5 members are also common to both sialomes, as are defensins. FS-I/Cys7 and the 8-Cys families of peptides are also shared by both fleas and are unique to these organisms. The Gly-His-rich peptide similar to holotricin was found only in the cat flea, as were the abundantly expressed Cys-less peptide and a novel short peptide family.

**Conclusions/Significance:**

Fleas, in contrast to bloodsucking Nematocera (mosquitoes, sand flies, and black flies), appear to concentrate a good portion of their sialome in small polypeptides, none of which have a known function but could act as inhibitors of hemostasis or inflammation. They are also unique in expansion of a phosphatase family that appears to be deficient of enzyme activity and has an unknown function.

## Introduction

Saliva of blood-feeding animals contains a mixture of compounds that prevent their host's physiologic defences against blood loss, or hemostasis, which is a complex response based on the functional triad of platelet aggregation, vasoconstriction, and blood clotting. Indeed, anticlotting, vasodilatory, and antiplatelet substances have been characterized from salivary gland (SG) homogenates of many ticks, blood-feeding insects, nematodes, annelids, and bats [1]–[5]. Hematophagous arthropod saliva may also contain antimicrobial compounds that might help to contain bacterial growth in the ingested blood bolus [1]. On the other hand, salivary proteins may generate irritating immune responses in their hosts that might be detrimental to blood feeding.

In the past 10 years, molecular biology advances allowed the description of organ-specific transcriptomes, obtained from the random DNA sequencing of clones derived from reverse transcription of organ-specific mRNA (which produces a DNA copy of the mRNA, or cDNA, the set of which is known as a cDNA library). Assembly of these random sequences and identification of their coding sequences (CDS) allows for the disclosure of sialotranscriptomes (from the Greek sialo  =  saliva). Accordingly, it is now possible to list 50 different proteins in sialomes of sand flies, while mosquitoes have nearly 100 putative secreted proteins, and ticks have several hundred [6], [7]. Most of these proteins have no known function, and many belong to protein families unique to the insect family or even genus, indicating a fast evolution of the coding genes, possibly due to the immune pressure imposed by hosts on their products.

**Figure 1:**
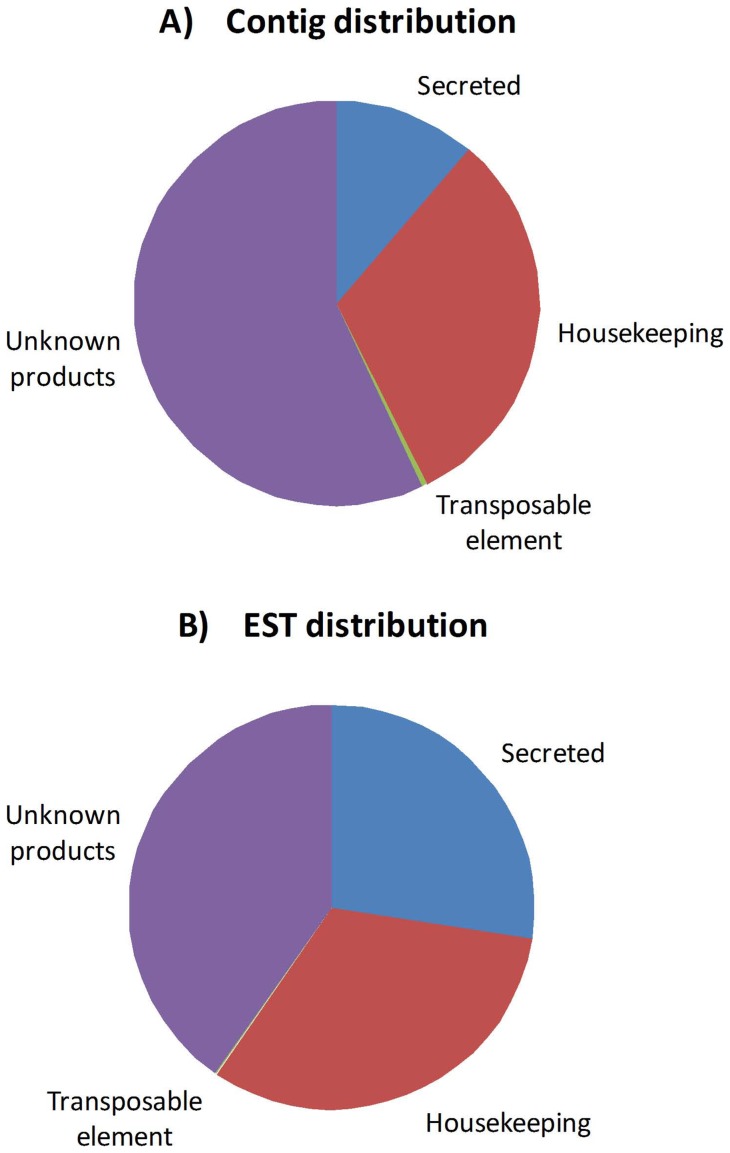
Cat flea sialotranscriptome. Distribution of assembled contigs (A) and number of expressed sequence tags (ESTs) (B) in the sialotranscriptome of the cat flea, *Ctenocephalides felis*.

The blood-feeding mode evolved independently among insects not less than ten times: at least twice in the true bugs (Heteroptera), five times in the flies (Diptera), and once each in lice (Anoplura), in fleas (Siphonaptera) and, exceptionally, in moths (Lepidoptera) [8]. While several sialotranscriptomes exist for members of the Diptera and Heteroptera, only one exists for fleas, namely for the rat flea *Xenopsylla cheopis* [9]. It is the purpose of this manuscript to explore the sialotranscriptome of the cat flea *Ctenocephalides felis*.

**Table 1:** Functional classification of the sialotranscriptome of the cat flea, *Ctenocephalides felis*.

Class	Number of contigs	Number of ESTs	EST's/Contig
**Putative secreted proteins**	**91**	**478**	**5.3**
Enzymes			
Phosphatase	21	81	3.9
Apyrase	3	13	4.3
5′-nucleotidase	2	2	1.0
Adenosine deaminase	4	16	4.0
Esterase	3	15	5.0
Antigen 5 family	3	15	5.0
Immunity related			
Attacin	1	3	3.0
Defensins	5	5	1.0
Flea -specific families			
FS-H family	19	42	2.2
Short peptide family	8	116	14.5
Abundant very short peptide family	7	124	17.7
FS-I antigen	1	5	5.0
Deorphanized flea proteins	4	17	4.3
Other putative secreted peptides	10	24	2.4
**Putative Housekeeping proteins**	**253**	**558**	**2.2**
Protein synthesis machinery	118	397	3.4
Unknown conserved	21	23	1.1
Proteasome machinery	9	17	1.9
Cytoskeletal	14	16	1.1
Metabolism, energy	15	15	1.0
Transcription machinery	13	15	1.2
Protein modification	12	14	1.2
Transporters	8	10	1.3
Storage	4	9	2.3
Signal transduction	8	8	1.0
Metabolism, carbohydrate	7	7	1.0
Protein Export	7	7	1.0
Metabolism, amino acid	6	6	1.0
Immunity related	3	4	1.3
Extracellular matrix	1	3	3.0
Metabolism, lipid	3	3	1.0
Nuclear Regulation	2	2	1.0
Metabolism, intermediary	1	1	1.0
Metabolism, nucleotide	1	1	1.0
**Transposable element**	**3**	**4**	**1.3**
**Unknown products**	**459**	**700**	**1.5**
**Total**	806	1740	

Fleas have the largest number of genera when compared to other orders of bloodsucking arthropods, indeed representing near half of all combined genera [3], [10]. It is believed that this large number of genera reflects the flea's co-speciation with their mammalian and bird hosts after dinosaur extinction and mammalian radiation, ∼65 million years ago (MYA). Indeed flea fossils have been recently described dating from the Mesozoic era, one specimen from the Jurassic (∼165 MYA), and another from the Lower Cretaceous period (∼125 MYA), far before the radiation of mammals [11]. Accordingly, the phylogenetic distance between the cat flea and rat flea should be not less than that separating cats and rats, dating to before the diversification of the Carnivora and that of Rodents and logomorphs on the Paleocene, over 60 MYA. It is thus not surprising that fleas could have as many genera as there are mammalian and bird genera.

**Figure 2:**
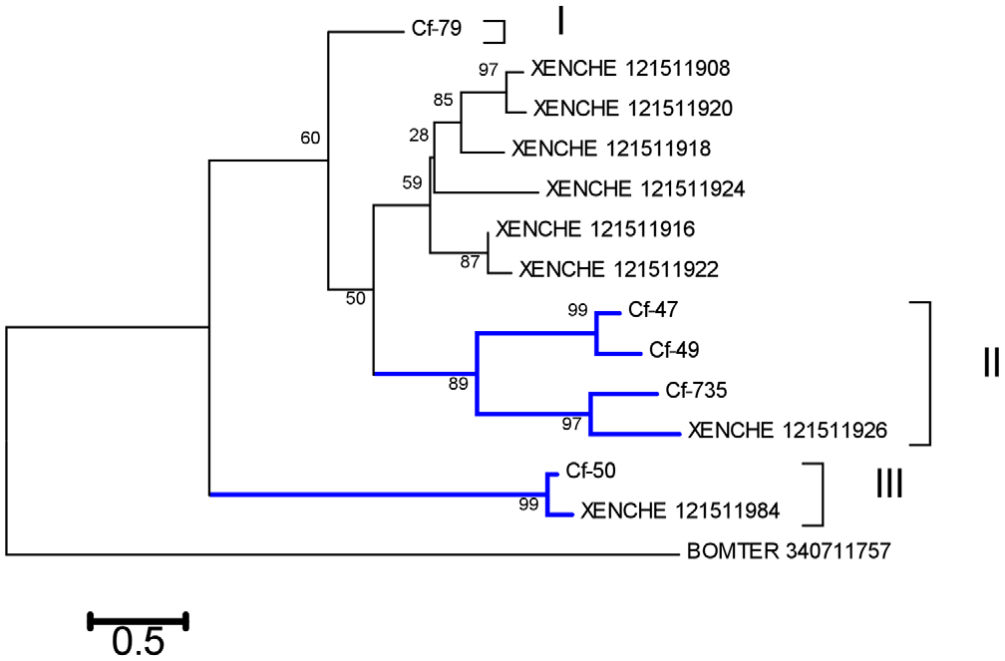
Phylogram of the flea salivary phosphatase family with one sequence from *Bombus terrestris* as an outgroup. The sequences were aligned by ClustalW. The *Ctenocephalides felis* sequences are recognized by starting with Cf- and are followed by the number of the contig from which they derived. The other sequences were obtained from GenBank and are recognized by the first three letters of their genus name, followed by the first three letters of the species name, followed by the NCBI accession number. The numbers at the nodes represent the percent bootstrap support (10,000 iterations) for the neighbor-joining algorithm, using pairwise deletion and gamma distribution of the amino acid substitutions. The bar at the bottom indicates the amino acid substitution rate per site. The Roman numerals indicate tree locations for the cat flea sequences that are distant enough to be from different genes. Clade II may have two genes, for a total of four possible genes.

SG homogenates of fleas have antiplatelet activity in the form of a platelet-activating factor (PAF) esterase [12] as well as apyrase activity that destroys ADP [9], [13], [14], an agonist of platelet aggregation released by injured cells and by activated platelets. Hyaluronidase activity was also detected in cat flea SGs [15]. This activity may help to spread other pharmacologically active salivary components into the host skin. Cat fleas can also cause important allergic reactions in cats, dogs, and humans [16]. Partial characterization of some of these antigens has been attempted [17]–[20], and a major antigen of 18 kDa from the cat flea, named Cte f1, has been identified [21]. Currently, there are only four salivary proteins deposited in GenBank, including the Cte f1 above mentioned (gi|4336703), which is identical to another deposited protein named FS-I (gi|3805687, which is a truncated form of Cte f1), an antigen 5 member (gi|7638032), and a peptide annotated as FS-H precursor (gi|1575479). Accordingly, there are only three salivary peptides known from *C. felis* that are publicly available. In contrast, the sialotranscriptome of *X. cheopis* identified an expanded phosphatase family of proteins (without a known function) as well as other enzymes including a CD-39 type of apyrase and an esterase; additionally, mucins, antimicrobial peptides, and members of the antigen 5 family were also described. Notably, one large family of peptides named the FS family, with >10 members (homologous to the *C. felis* FS-I protein) was identified, together with 15 other peptides of novel families. Here we report on the sialotranscriptome of the cat flea, *C. felis.*

**Figure 3:**
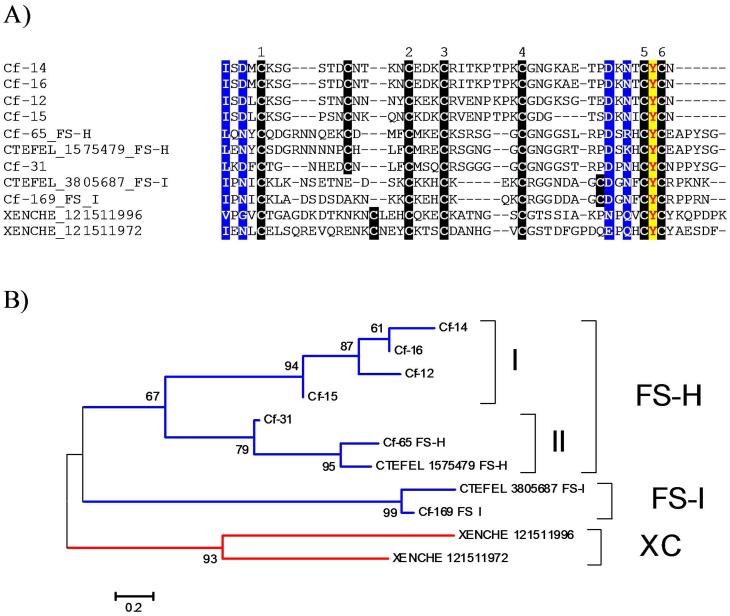
The FS-H/FS-I/7-Cys family of flea salivary peptides. (A) ClustalW alignment indicating the cysteine residues in black background, the identical Tyr in yellow background, and the conserved amino acids in blue background. The numbers above the sequence indicate the six conserved cysteines. The signal peptide region is not shown. (B) Bootstrapped phylogram of the sequences based on the alignment in (A) after 1,000 iterations. The numbers at the nodes indicate the percent bootstrap support, and the bar at the bottom the amino acid divergence per site. Sequences identified in this work are named Cf- followed by the number of the originating contig from File S1. Sequences derived from GenBank are recognized by the first three letters of their genus name, followed by the first three letters of the species name, followed by the gi| accession number. The cat flea proteins giving the name of the family are indicated by FS-H and FS-I following their accession numbers.

**Figure 4:**
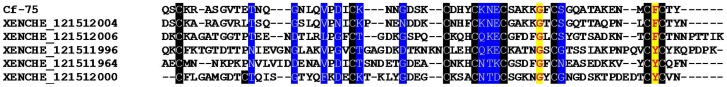
The deorphanized 8-Cys family of flea salivary peptides. (A) ClustalW alignment indicating the cysteine residues in black background, the identical Gly as well as the conserved Phe and Tyr in yellow background, and remaining conserved amino acids in blue background. The numbers above the sequence indicate the eight conserved cysteines. The signal peptide region is not shown. The sequences identified in this work are named Cf- followed by the number of the originating contig from File S1. The sequences derived from GenBank are recognized by the first three letters of their genus name, followed by the first three letters of the species name, followed by the gi| accession number.

## Methods

### Flea Salivary Gland (SG) Preparation

Unfed adult *C. felis* were purchased from Elward II (Soquel, CA, USA). Multiple generations of adult fleas were provided a bovine blood meal via an artificial dog [22], and eggs were reared to adults on sand with artificial diet [23] at Louisiana State University (Baton Rouge, LA, USA). For tissue collection, newly emerged adult fleas were fed bovine blood for 7 days. Twenty pairs of SGs were extracted from fleas daily starting on day 0 (unfed). Briefly, fleas were immobilized on ice and dissected by standard mircodissection techniques. SGs were immediately placed into RNAlater (Ambion, Inc., Austin, TX, USA) and stored at 4°C until used for RNA extraction.

**Figure 5:**
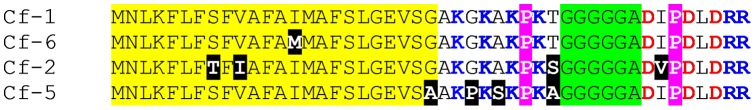
The cat flea Cys-less peptide family. ClustalW alignment showing the non-conserved residues in black background and the signal peptide in yellow background. Positively charged amino acids on the mature peptide are shown in blue color; acidic residues are shown in red. The GGGGGA motif is shown in green background. The conserved prolines flanking the glycine-rich motif are shown in pink background.

**Figure 6:**
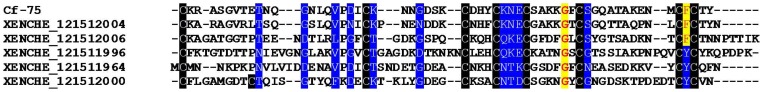
The short flea salivary peptide. ClustalW alignment indicating the cysteine residues in black background, the identical amino acids in yellow background, and the conserved amino acids in blue background. The signal peptide region is not shown. The sequences identified in this work are named Cf- followed by the number of the originating contig from File S1. The sequences derived from GenBank are recognized by the first three letters of their genus name, followed by the first three letters of the species name, followed by the gi| accession number.

### Library Construction

SG RNA, extracted from 160 pairs of intact glands, was isolated using the Micro-FastTrack mRNA isolation kit (Invitrogen, San Diego, CA, USA). Other procedures were as described before [24], [25] and are reproduced here for easiness of access to the reader:

“The PCR-based cDNA library was made following the instructions for the SMART (switching mechanism at 5′end of RNA transcript) cDNA library construction kit (Clontech, Palo Alto, CA, USA). This system uses oligoribonucleotide (SMART IV) to attach an identical sequence at the 5′ end of each reverse-transcribed cDNA strand. This sequence is then utilized in subsequent PCR reactions and restriction digests.

First-strand synthesis was carried out using MMLV (Maloney murine leukemia virus) reverse transcriptase (Clontech) at 60°C for 1 h, then at 42°C for 40 min in the presence of trehalose and the SMART IV and CDS III (3′) primers. Second-strand synthesis was performed by a long-distance PCR-based protocol using Advantage Taq polymerase mix (Clontech) in the presence of the 5′ PCR primer and the CDS III (3′) primer. The cDNA synthesis procedure resulted in creation of *SfiI A* and *B* restriction enzyme sites at the ends of the PCR products that are used for cloning into the phage vector (λ TriplEx2 vector; Clontech). PCR conditions were as follows: 95°C for 1 min; 22 cycles of 95°C for 15 sec, 68°C for 6 min. A small portion of the cDNA obtained by PCR was analyzed on an E-Gel® 1.2% agarose/EtBr (Invitrogen) to check quality and range of cDNA synthesized. Double-stranded cDNA was immediately treated with proteinase K (0.8 μg/mL) at 45°C for 20 min, and the enzyme was removed by ultrafiltration through a Microcon YM-100 centrifugal filter device (Amicon Inc., Beverly, CA, USA). The cleaned double-stranded cDNA was then digested with *SfiI* restriction enzyme at 50°C for 2 h, followed by size fractionation on a ChromaSpin–400 drip column (Clontech) into small (S), medium (M), and large (L) transcripts based on their electrophoresis profile on an E-Gel® 1.2%agarose/EtBr. Selected fractions were pooled and concentrated using a Microcon YM-100.

The concentrated cDNA mixture was ligated into the λ TriplEx2 vector, and the resulting ligation mixture was packaged using the GigaPack® III Plus packaging extract (Stratagene, La Jolla, CA, USA) according to the manufacturer's instructions. The packaged library was plated by infecting log-phase XL1-Blue *Escherichia coli* cells (Clontech). The percentage of recombinant clones was determined by blue-white selection screening on LB/MgSO_4_ plates containing X-gal/IPTG. Recombinants were also determined by PCR, using vector primers PT2F1 (AAG TAC TCT AGC AAT TGT GAG C) and PT2R1 (CTC TTC GCT ATT ACG CCA GCT G) flanking the inserted cDNA, with subsequent visualization of the products on an E-Gel® 1.2% agarose/EtBr.”

### cDNA Sequencing

This was done as described before [24], [25] and is reproduced here for easiness of access to the reader:

“Twenty 96-well plates were prepared for cycle sequencing, each containing 94 clones and two DNA controls, as follows: The cDNA library was plated on LB/MgSO_4_ plates containing X-gal/IPTG to an average of 250 plaques per 150 mm Petri plate. Recombinant (white) plaques were randomly selected and transferred to 96-well microtiter plates (Nunc, Rochester, NY, USA) containing 75 μL of ultrapure water (KD Medical, Columbia, MD, USA) per well. The phage suspension was either immediately used for PCR or stored at 4°C for future use.

To amplify the cDNA using a PCR reaction, 5 μL of the phage sample was used as a template. The primers were sequences from the λ TriplEx2 vector and named PT2F1 (AAG TAC TCT AGC AAT TGT GAG C) and PT2R1 (CTC TTC GCT ATT ACG CCA GCT G), positioned at the 5′ end and the 3′ end of the cDNA insert, respectively. The reaction was carried out in a 96-well PCR microtiter plate (Applied Biosystems, Inc., Foster City, CA, USA) using FastStart Taq polymerase (Roche Diagnostics, Mannheim, Germany) on a GeneAmp PCR system 9700 (Perkin Elmer Corp., Foster City, CA, USA). The PCR conditions were 1 hold of 75°C for 3 min; 1 hold of 94°C for 4 min, 30 cycles of 94°C for 1 min, 49°C for 1 min; 72°C for 4 min. Amplified products were analysed on an E-Gel® 1.2% agarose/EtBr. Clones were PCR amplified, and those showing a single band were selected for sequencing. Approximately 200–250 ng of each PCR product was transferred to a 96-well PCR microtiter plate (Applied Biosystems) and frozen at –20°C. Samples were shipped on dry ice to the Rocky Mountain Laboratories Genomics Unit (NIAID, NIH, Hamilton, MT, USA) with primer (PT2F3: TCT CGG GAA GCG CGC CAT TGT) and template combined together in a 96-well optical reaction plate (P/N 4306737; Applied Biosystems) following the manufacturer's recommended concentrations. Sequencing reactions were set up as recommended by Applied Biosystems' BigDye® Terminator v3.1 cycle sequencing kit by adding 1 μL ABI BigDye® Terminator ready reaction mix v3.1 (P/N 4336921), 1.5 μL 5x ABI sequencing buffer (P/N 4336699), and 3.5 μL of water for a final volume of 10 μL. Cycle sequencing was performed at 96°C for 10 sec, 50°C for 5 sec, 60°C for 4 min for 27 cycles on either a Bio-Rad Tetrad 2 (Bio-Rad Laboratories, Hercules, CA. USA) or ABI 9700 thermal cycler (Applied Biosystems). Fluorescently labeled extension products were purified following Applied Biosystems'BigDye® XTerminator™ purification protocol and subsequently processed on an ABI 3730xL DNA Analyzer (Applied Biosystems).”

The coding sequences described in this work were deposited to NCBI's GenBank with accessions JW050188-JW050244.

### Bioinformatics Tools and Procedures

This was done as described before [24], [25] and is reproduced here for easiness of access to the reader:

“Expressed sequence tags (EST) were trimmed of primer and vector sequences. The BLAST tool [26], CAP3 assembler [27] and ClustalW [28] software were used to compare, assemble, and align sequences, respectively. Phylogenetic analysis and statistical neighbor-joining bootstrap tests of the phylogenies were done with the Mega package [29]. For functional annotation of the transcripts, we used the tool blastx [26] to compare the nucleotide sequences to the NR protein database of the NCBI [30] and to the Gene Ontology (GO) database [31]. The tool, reverse position-specific BLAST (rpsblast) [26] was used to search for conserved protein domains in the Pfam [32], SMART [33], Kog [34], and conserved domains (CDD) databases [35]. We also compared the transcripts with other subsets of mitochondrial and rRNA nucleotide sequences downloaded from NCBI. Segments of the three-frame translations of the ESTs (because the libraries were unidirectional, six-frame translations were not used), starting with a methionine found in the first 300 predicted amino acids (AAs), or the predicted protein translation in the case of complete CDS, were submitted to the SignalP server [36] to help identify translation products that could be secreted. O-glycosylation sites on the proteins were predicted with the program NetOGlyc [37]. Functional annotation of the transcripts was based on all the comparisons above. Following inspection of all these results, transcripts were classified as either secretory (S), housekeeping (H), or of unknown (U) function, with further subdivisions based on function and/or protein families. Putative sequences deriving from transposable elements (TE) were also found.”

## Results and Discussion

### Overall Transcriptome Assembly and Annotation

A total of 1,740 ESTs were assembled into 806 contigs, including singletons (see spreadsheet S1). Of these, 91 contigs are predicted to code for putative secreted proteins that may be constituents of the flea saliva (S class), with an average of 5.3 ESTs per contig. This S class contains 28% of the ESTs and 11% of the contigs. Five hundred fifty eight ESTs (32% of total ESTs) assembled into 253 contigs that are classified as coding for housekeeping proteins (H class), with an average of 2.2 ESTs/contig. The H class is presumed to encompass those transcripts associated with the maintenance of the cells, including protein synthesis, but not be coding for constituents of the salivary secretion. The H class contains 32% of the ESTs and 31% of the contigs. We could not predict the function of 700 ESTs assembled into 459 contigs, representing 40% of the ESTs. Finally, 4 contigs deriving from 3 ESTs code for sequences similar to TEs, a common finding in sialotranscriptomes (Table 1 and Figure 1). This transcriptome EST and contig distribution contrasts with that found for the rat flea sialotranscriptome [9], where 75% of the ESTs were classified as belonging to the S class, nearly 3 times the value found here. Each contig can be found in File S1, which is an annotated spreadsheet having links to sequence comparisons in several databases.

From the assembled contigs found in File S1, open reading frames were identified and protein sequences were deposited in File S2, another hyperlinked spreadsheet. The remaining subtitles of this section are a guide for browsing these two spreadsheets.

### Putative Secreted Proteins

Enzymes, members of the antigen-5 protein family, immune-related peptides and flea-specific families of unknown function are identified as putative secreted polypeptides in the sialotranscriptome of the cat flea. These classes are further described below.

#### Enzymes

Phosphatases, apyrase of the CD39 family, adenosine deaminase, and esterases were identified. These enzyme sequences share similarities to those found in the rat flea sialotranscriptome [9].

#### Phosphatases

The phosphatase family in the cat flea is represented by 81 ESTs, or nearly 17% of all ESTs of the S class. Alignment of translated phosphatase protein sequences from the cat flea with those of the rat flea and a sequence from *Bombus terrestris* as an outgroup (Figure 2) shows the diversity of this family, with possibly four related genes being involved in the production of the *C. felis* transcripts, two of which are on clade II (Figure 2). The identity between rat and cat flea phosphatases varies from 21 to 84%, indicating the divergence between these salivary proteins among different flea genera.

#### Apyrases, 5′ nucleotidases, and adenosine deaminases

Apyrase of the CD-39 family, 5′ nucleotidases, and adenosine deaminase-coding transcripts were found in the cat flea sialotranscriptome, similarly to the rat flea [9], indicating an active purinergic degradation pathway all the way from ATP to inosine, NH_3_, and phosphate, as is found in *Aedes* and *Culex* mosquitoes [38] and also in sand flies [39]–[41]. It is interesting to note that these protein sequences are at best 60% identical in primary sequence to their best match deriving from rat fleas, indicating considerable divergence between these related proteins.

#### Esterases

Truncated esterase-coding transcripts were identified, producing best matches by blastp to their homologs from rat fleas varying from 37 to 56% identity at the amino acid level. These derive from at least two different genes, because the deducted protein sequences are less than 60% identical between pairs.

#### Antigen-5 family

This is a ubiquitous protein family found in wasp and snake venoms as well as in virtually all arthropod sialotranscriptomes done so far. Most of these proteins have no known function, but in snakes it was associated with channel-blocking activities [42].

#### Antimicrobial peptides

A typical **defensin**, deducted from a singleton, was identified in the sialotranscriptome of *C. felis*. It has the Defensin_2 domain of the PFAM database and matches several insect proteins annotated as defensins in the NR, Swissprot, and GO databases. Another CDS, assembled from 9 ESTs, codes for a Gly- and His-rich peptide and is 55% identical to holotricin-3 in its primary structure. Holotricins are antimicrobial peptides ∼ 100 AAs long previously identified from the beetle *Holotrichia diomphalia* [43]. Antimicrobial peptides are a common finding in the sialotranscriptomes of hematophagous arthropods, where it may help to subdue microbial growth in the blood meal as well as to contain infection in their host's feeding lesions.

#### FS-H/FS-I antigen/7-Cys family of flea-specific peptides

FS-H and FS-I antigens refer to proteins deposited in GenBank that were identified as flea antigen candidates in a previous study [44]. Homologs from the rat flea were also identified previously [9]. Seven members of this family were additionally recognized in the present study (Figure 3), assembled from 4 to76 ESTs each. No identical match to the previously identified cat flea peptides were found, the closest matches having 73 to 76% identity at the primary structural level only (File S2 and Figure 3A). Alignment of the flea sequences recognizes a framework of six conserved cysteines, possibly involved in three disulphide bonds, plus one odd cysteine that might be involved in redox reactions (Figure 3A). The odd cysteine in the FS-I subfamily is in a different position when compared with other family members. A conserved Cys-Tyr-Cys triplet is found in the carboxyterminus, plus a few sites with conserved AA residues (Figure 3A). Phylogenetic analysis indicates three robust clades, one containing the FS-H sequence, another containing the FS-I, and the third having the rat flea sequences (Figure 3B). The FS-H clade further divides into two subclades, each containing three and four sequences. The analysis indicates that at least three genes code for this protein family in the cat flea, if we consider a divergence of 20% in the AA identity per site as a cut-off to differentiate alleles from genes. The function of this protein family is unknown, but it may be acting as an antioxidant as occurs with other proteins having unpaired cysteines, such as plasma α-microglobulin [45] or frog skin antioxidant peptides [46].

#### Deorphanized 8-cys flea peptide family

The peptide encoded by Cf-75 (File S2), assembled from 14 ESTs, has 33–66% identity to a rat flea salivary peptide family that had no significant similarities to other peptides found in GenBank, thus deorphanizing this protein family. Alignment of Cf-75 with four rat flea sequences shows a conserved framework of eight cysteines (Figure 4), including a triad of Cys-[Phe/Tyr]-Cys at the carboxyterminus, which is similar to the Cys-Tyr-Cys triad of the FS-H/FS-I antigen/7-Cys family of flea-specific peptides presented above. It is possible that the 8-Cys family is thus related to the 7-Cys family despite poor conservation of other residues. The function of any member of this family remains unknown.

#### Cys-less short peptide family

Over 90 ESTs assembled into 6 contigs coding for short peptides of mature MW of 2.3 kDa containing 23 AAs, without cysteines. Figure 5 shows four such sequences that were assembled by 10 to 42 ESTs each. Notice that there are only a few AA differences between the sequences, indicating that these could derive from a polymorphic gene or from closely related genes. The mature peptide has three clearly distinguished domains: a basic region with alternating apolar and AAs, a glycine-rich middle part, and an acidic-rich carboxyterminus that ends in two arginines. The glycine-rich domain is flanked by conserved proline residues that might give some structure to the peptide. These peptides do not produce significant matches when compared to the NR database. The function of this peptide family is unknown.

#### Another short flea peptide

The assembly of eight ESTs provided for a contig coding for a putative secreted 36 amino acid long peptide encoded by Cf-25 (File S2) containing a single Cys near the amino terminal region (Figure 6). This peptide has no significant matches to proteins deposited in the NR database.

### Putative housekeeping proteins

Several contig sequences match proteins functionally identified as housekeeping, most belonging to the protein synthesis machinery (397 of the 558 ESTs on the H class), as expected for the nature of the organ (Table 1). Extracted CDS, mostly for ribosomal proteins, are included in File S2.

### Comparisons between rat and cat flea protein sequences

From the standpoint of protein families that appear to be secreted, the sialomes of both cat and rat fleas have the following enzyme families: phosphatases, CD-39-type apyrase, adenosine deaminases, and esterases. Antigen-5 members are also common to both sialomes, as are defensins. The FS-I/Cys7 and the 8-Cys families of peptides, unique to fleas, are also shared by both fleas. The Gly-His rich peptide similar to holotricin, assembled from nine ESTs, was found only in the cat flea. Also unique to the cat flea, the abundantly expressed (>90 ESTs) Cys-less peptide–as well as another short peptide family–underscores the fast evolution of salivary proteins in bloodsucking arthropods. The rat flea sialome also presents unique peptides, including the short peptide encoded by gb|ABM55436.1|, which also has the dipolarity of acid and basic residues described for the Cys-less peptide of the cat flea but no similarities in primary structure and, indeed, the order of the polar AAs are reversed. Several other rat flea peptides with no similarity to the presently described cat flea sialome also exist, emphasizing the diversity of the sialome of hematophagous insects even at the genus level.

Comparison of 16 housekeeping sequences best matching *X. cheopis* sequences deposited on the NR database from our previous study [9] shows an average sequence identity of 95% ±3.5%, while 18 sequences of the S class best matching *X. cheopis* sequences have only 47% ±13.7% sequence identity (average ± SD). These results are significant–with a P<0.001 when tested by the *t*-test with correction for unequal variances–and are another indication that salivary proteins are under a fast pace of evolution, as indicated before for mosquitoes and ticks [1].

## Supporting Information

File S1 **Hyperlinked Excel spreadsheet containing annotated assembled ESTs.** (S1): http://exon.niaid.nih.gov/transcriptome/C_felis/Cf-S1.zip.
(XLSX)

File S2 **Hyperlinked Excel spreadsheet containing annotated coding sequences.** (S2): http://exon.niaid.nih.gov/transcriptome/C_felis/Cf-S2.zip.
(XLSX)
